# Correlation of baseline hormonal disorders with immunological failure and mortality in male HIV patients during follow-up

**DOI:** 10.1097/MD.0000000000005732

**Published:** 2016-12-30

**Authors:** Ying Wen, Hai bo Ding, Wei Chen, Ying Zhou, Wen Wang, Yu Wang, Xu Lu, Jing Liu, Jing Kang, Wenqing Geng, Hong Shang, Pei Liu

**Affiliations:** a Department of Infectious Diseases, The First Affiliated Hospital; b Department of Laboratory Medicine, Key Laboratory of AIDS Immunology of National Health and Family Planning Commission, The First Affiliated Hospital, China Medical University, Shenyang, Liaoning Province; c Collaborative Innovation Center for Diagnosis and Treatment of Infectious Diseases, Hangzhou; d Shenyang Chest Hospital, Shenyang, Liaoning Province, China.

**Keywords:** endocrine disorders, human immunodeficiency virus 1, immunological failure, mortality

## Abstract

To assess the effect that hormonal disturbances have on HIV prognosis in male patients. A prospective follow-up study was conducted among male HIV patients who started antiretroviral therapy (ART) between July 1, 2011 and June 30, 2014. The final follow-up session occurred before December 31, 2014. We examined the correlation between pre-ART hormone levels and disease prognosis. The Kaplan–Meier method and the multivariate Cox proportional hazard model were used to identify hormone-related predictors of immunological failure and mortality. During the follow-up of 163 male HIV patients, mortality rate occurred at a rate of 16.0% (26/163). Of these deaths, 84.6% (22/26) were acquired immunodeficiency syndrome–related. Furthermore, 53 patients were found to have suffered from immunological failure. Both pre-ART CD4+ T cell counts and the clinical stage assigned to the patients correlated strongly with dehydroepiandrosterone sulfate levels. Hyponatremia, high cortisol levels, tuberculosis, and being at World Health Organization (WHO)-defined clinical stage 4 were characteristics that associated significantly with mortality. Being at WHO clinical stage 4 was, itself, a factor that significantly associated with immunological failure. High cortisol levels were found to be an important hormonal disorder that associated with mortality. None of the hormones examined in this study had a strong correlation with immunological failure.

## 1 Introduction

Multiple endocrine abnormalities that have subclinical or overt clinical features have been reported among patients infected with human immunodeficiency virus 1 (HIV-1).^[1]^ In the advanced stages of HIV infection, severe HIV-associated immunosuppression and multiple opportunistic infections (OIs) have been implicated in the etiology of these endocrine abnormalities.^[2]^ Medications used to manage HIV infection can also contribute to endocrine abnormalities. Hypogonadism is the most common endocrine dysfunction, followed by thyroid and adrenal dysfunctions. Notably, hypocortisolism is not common, but high cortisol levels may be a marker of stress due to HIV infection or other associated infections.^[3]^ Studies of cortisol reveal that higher serum cortisol levels are associated with a faster progression from HIV infection to acquired immunodeficiency syndrome (AIDS).^[4]^ A hypercortisolemic state may hasten HIV-1 disease progression by stimulating viral replication, modifying programed cell death, altering the secretion pattern of cytokines, and suppressing T helper cell-directed immunity.^[5–7]^ Studies disagree on the nature of the correlation between CD4+ T cell counts and the occurrence of thyroid dysfunction and hypogonadism.^[8–11]^ However, more importantly, we are yet to discover a definitive explanation for why some patients who respond well to antiretroviral therapy (ART) experience an unexplained drop in CD4+ T cell counts. In addition, very few published studies correlate endocrine dysfunction with immunological failure and mortality.^[12]^ Apart from the hormones mentioned above, other hormonal problems, especially those that correlate with clinical impact, are rarely studied. In our previous study, we proposed that inappropriate secretion of antidiuretic hormone (ADH) (SIADH) was a major cause of persistent hyponatremia in HIV patients.^[13]^ Moreover, renin could enhance HIV replication in T cells in a dose-dependent manner.^[14]^ In HIV patients, brain natriuretic peptide (BNP) is helpful for assessing body fluid volumes and is also a valuable marker of cardiac dysfunction.^[15]^ This article prospectively evaluates the hormonal profile of HIV patients and focuses on how hormones may affect disease prognosis.

## 2 Methods

### 2.1 Selection of patients

We performed a prospective analysis on male patients who were newly diagnosed with HIV infection and had begun ART between July 1, 2011 and June 30, 2014 at the First Affiliated Hospital of China Medical University (Shenyang, China). The final follow-up session occurred before December 31, 2014. Patients who had a history of sexually transmissible infections were recruited, but intravenous drug users and former plasma donors were excluded. Patients who were 16 years or older were recruited, and children younger than 16 years and women were excluded. Patients who had used corticosteroids within the month immediately preceding recruitment were excluded. Patients who refused to receive ART or did not meet the criteria for receiving ART for free were excluded. ART is offered for free to patients if their OIs are well managed and they received regular follow-ups at the clinic run by the Key Laboratory of AIDS Immunology of the Ministry of Health at the First Affiliated Hospital of China Medical University. If complications occurred, patients could choose to be admitted to the Department of Infectious Diseases for treatment. Routine monitoring of CD4+ T cell counts was performed every 6 months, or more frequently if clinically indicated. Patients who had had at least 2 follow-ups were recruited. The control group consisted of 80 males, age-matched, healthy volunteers. Patients who were lost to follow-up were considered to be “censored.”

This study was approved by the Clinical Research Ethics Committee of China Medical University ([2011]36), and informed consent was obtained from all participants.

### 2.2 Definitions

Free ART was offered to patients whose CD4+ T cell counts were less than 350 cells/μL or were categorized as World Health Organization (WHO) stage 4 irrespective of CD4+ T cell count. Hyponatremia is defined as serum sodium concentrations <135 mmol/L. Cytomegaloviremia is defined as Cytomegalovirus (CMV) Deoxyribonucleic acid (DNA) ≥ 250 copies/mL. Immunological failure is defined according to the WHO criteria for ART (2010 revision) to be applied to HIV infection in adults and adolescents and that were used as public health recommendations. These criteria are a decrease in CD4+ T cell counts to pre**-**ART levels or below, a decrease in CD4+ T cell counts by more than 50% of on-treatment peak values, or persistent CD4+ T cell counts of <100 cells/μL after 6 months of therapy.

Subjects were allowed to miss no more than 1 dose of once-daily treatments in the preceding month, or no more than 3 doses of twice-daily treatments in the preceding month. Despite these restrictions, the study experienced an adherence rate of more than 95% of patients. Virological failure was defined as HIV-Ribonucleic acid (RNA) > 1000 copies/mL after 6 months of ART.

### 2.3 Study design and assessment of hormonal disorders

In order to assess the levels of the 7 aforementioned hormones, blood samples were obtained between 1 and 7 days before the start of ART. After centrifugation, plasma aliquots were preserved at −80 °C. Serum cortisol levels were measured at 8 am. Levels of serum cortisol, adrenocorticotropic hormone (ACTH), BNP, supine aldosterone, ADH, renin, and dehydroepiandrosterone sulfate (DHEA-S) were detected using an Enzyme Linked Immunosorbent Assay Kit, provided by Shanghai Westang Bio-Tech Co., Ltd., Shanghai, China.

Clinical data, including demographic data—such as age, body mass index (BMI, kg/m^2^), underlying medical conditions, clinical presentation, and clinical course—were obtained from patients’ medical records. Laboratory tests were reviewed by a trained team of physicians, and results were entered into a computerized database. We also analyzed leukocyte count, polymorphonuclear cell count, hemoglobin levels, platelet count, glutamic alanine transaminase levels, total bilirubin, serum albumin levels, prothrombin time, serum sodium concentration, serum creatinine, C-reactive protein (CRP), CD4+ T cell counts, and HIV-RNA load.

Immunological failure and death were considered to be endpoint events. Factors that might influence immunological failure and long-term survival were analyzed.

### 2.4 Statistical methods

Continuous normal variables were represented as “median (interquartile range),” and categorical variables were expressed as a percentage of the number of cases. HIV-RNA levels (copies/mL) were log_10_-transformed into variables (log_10_ copies/mL). Mean values of these continuous variables were compared using the Student *t* test for data that distributed normally. Otherwise, the Mann–Whitney *U* test was used. Proportions related to the categorical variables were compared using the chi-squared test, although Fisher exact test was used when the data were sparse. Pearson correlation was used to evaluate the relationship between CD4+ T cell counts or HIV-RNA load and serum hormone concentrations. The Student *t* test or the Mann–Whitney *U* test was used to analyze the association between serum hormone concentrations and clinical WHO stage 4 categorization. The Kaplan–Meier method and the multivariate Cox proportional hazard model were employed to identify predictors of immunological failure and mortality. Data with a *P* value <0.1 and that lacked collinearity were entered into the multivariate Cox proportional hazard model. The hazard ratio (HR) was computed with a 95% confidence interval (CI), and *P* values <0.05 were considered to be statistically significant for all cases. All analyses were performed by using SPSS software for Windows version 17.0 (Chicago, IL).

## 3 Results

### 3.1 Clinical and laboratory test characteristics, coinfection, and cotreatment

A total of 219 hospitalized male patients who were newly diagnosed with HIV infection began receiving ART between July 1, 2011 and June 30, 2014 at the First Affiliated Hospital of China Medical University. However, 56 patients did not meet the study criteria, including 9 patients who declined to participate and 2 patients who had received only 1 follow-up.

The median age of the 163 eligible participants was 39 (age range: 21–75) years, and 108 (66.3%) patients were men who have sex with men. Only 15 patients had underlying medical conditions, including cirrhosis (2), hypertension (5), a history of tuberculosis (5), and diabetes (3). The cigarette index (number of cigarettes smoked per day × years of smoking) was higher than 400 for 17 patients. Three patients were lost to follow-up. Among 163 eligible patients, the following comorbidities were reported: oral or esophageal candidiasis (n = 65, 39.9%), hepatitis B (n = 15, 9.2%), hepatitis C (n = 11, 6.7%), pneumocystis pneumonia (PCP) (n = 34, 20.9%), tuberculosis (n = 64, 39.3%), central nervous system disease (n = 21, 12.9%), malignant tumors (n = 8, 4.9%), bacterial infections (n = 14, 8.6%), and cytomegaloviremia (n = 54, 33.1%). According to WHO definitions, 35 of our HIV-positive patients were at clinical stages 1 or 2; 32 patients were at clinical stage 3; and 96 patients were at clinical stage 4. In this study, the patients were grouped according to their CD4+ T cell counts: <100 cells/μL (n = 87, 53.4%), 100 to 200 cells/μL (n = 26, 16.0%), and 201 to 349 cells/μL (n = 50, 30.7%). The patients were also grouped according to their HIV-RNA levels: ≥10^5^ copies/mL (n = 85, 52.1%) and <10^5^ copies/mL (n = 78, 47.9%) (Table 1).

**Table 1:**
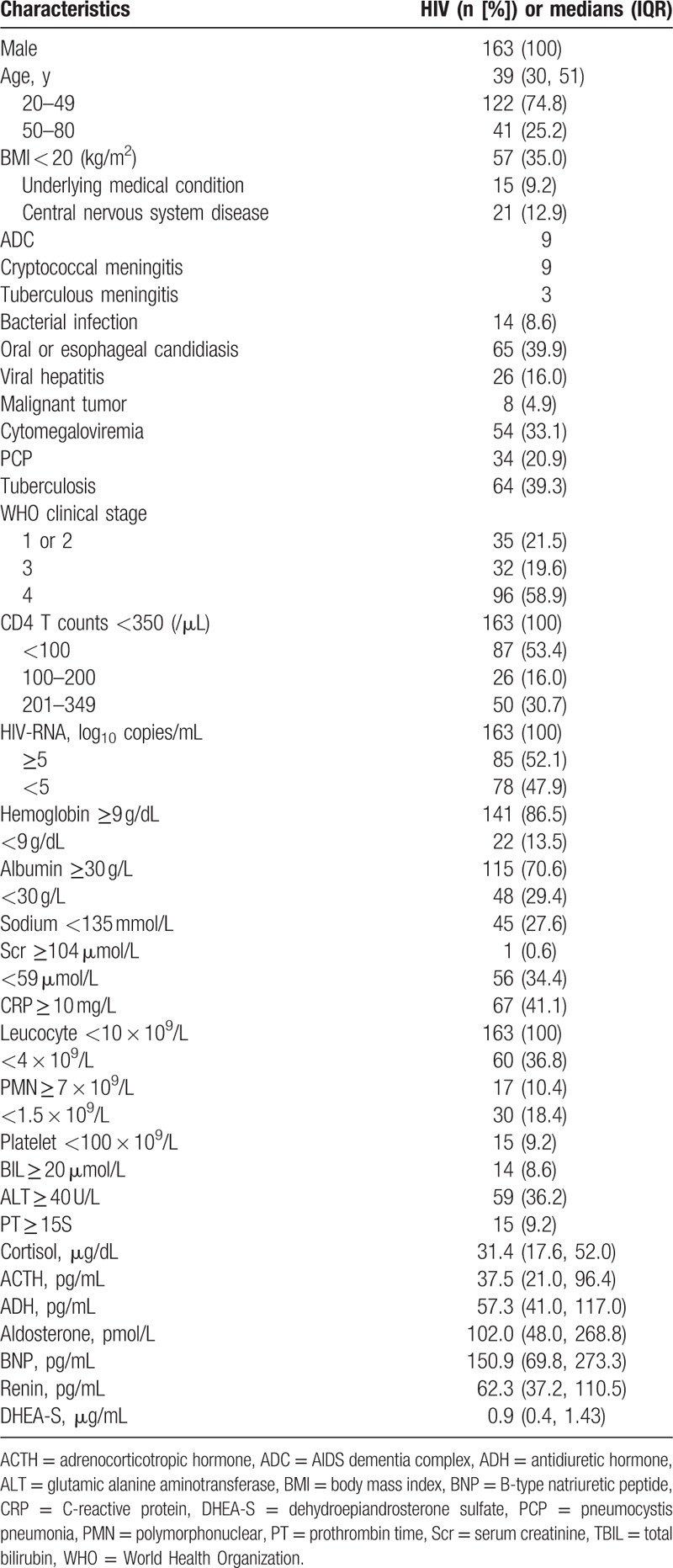
Baseline clinical characteristics and laboratory abnormities in HIV patients.

Patients received treatment for severe OIs before receiving ART. Zidovudine (or tenofovir), lamivudine, and efavirenz are the first choices for ART regimens. Patients with active tuberculosis were given first-line tuberculosis drugs recommended by the WHO. Eight patients who had latent tuberculosis infections received isoniazid preventive therapy. Patients who had CD4+ T cell counts less than 200 cells/μL received trimethoprim-sulphamethoxazole preventive therapy, except the 12 patients who were allergic to the drug. Patients with cryptococcal meningitis were treated with amphotericin B (or fluconazole) in combination with flucytosine followed by fluconazole therapy. Patients with cytomegaloviremia had undergone preemptive treatments with ganciclovir or foscarnet. Patients with hepatitis C did not receive interferon-α and ribavirin until their CD4+ T cell counts were higher than 350 cells/μL. Patients with hepatitis B received tenofovir-containing ART. The adherence rate of 160 patients was higher than 95%. A total of 21 patients changed ART regimens because of drug side effects (6 patients), drug interactions (5 patients), and drug resistance (10 patients). Lopinavir/Ritonavir was the most accessible second-line drug.

### 3.2 Correlation analysis between concentrations of 7 plasma hormones and baseline characteristics

The HIV patients were similar in age and BMI (*t* = −0.596, *P* = 0.552; *t* = 1.917, *P* = 0.056) to the healthy subjects. However, plasma cortisol, ACTH, BNP, and renin concentrations in the HIV patients were significantly higher, and DHEA-S levels were significantly lower than those of the healthy subjects (Table 2).

**Table 2:**
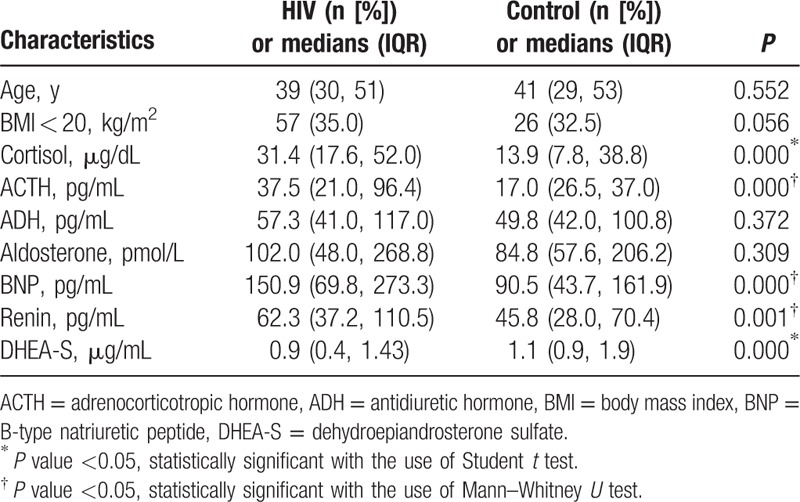
Data in HIV patients compared with healthy subjects (medians [IQR]).

Pearson correlation analysis suggested that only serum DHEA-S concentration correlated positively with CD4+ T cell counts (*r* = 0.422, *P* = 0.000). In addition, none of the 7 hormone concentrations correlated with HIV-RNA load. The Student *t* test suggested that patients at WHO stage 4 had lower serum DHEA-S levels compared with patients at other WHO stages (WHO clinical stages 1, 2, or 3) (*t* = 5.836, *P* = 0.000).

### 3.3 Risk factors for immunological failure

**Figure 1:**
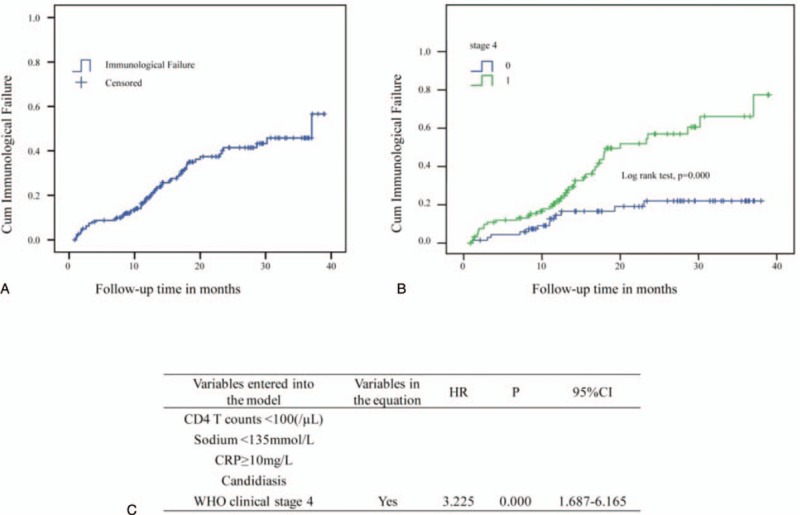
Kaplan–Meier curve and Cox regression for immunological failure of human immunodeficiency virus (HIV) patients undergoing antiretroviral therapy (ART). (A) Trend of cumulative immunological failure of patients within 39 months of ART follow-up. (B) Being at World Health Organization clinical stage 4 has a significant effect on immunological failure. (C) Factors associated with immunological failure among HIV patients by multivariate Cox regression analysis.

Study participants were followed for a minimum of 0.87 months and a maximum of 38.97 months with a total person-time follow-up of 228.71 person-years (PY). The rate of immunological failure was 32.5% (53/163). According to the WHO criteria, among the patients who experienced immunological failure, 27 (50.9%) immunological failures were marked by a decrease in CD4+ T cell counts to pre-ART levels or below (5 patients experienced confirmed virological failure), 10 (18.9%) failures were marked by CD4+ T cell counts that experienced a >50% decrease from on-treatment peak values (5 patients experienced confirmed virological failure), 16 (30.2%) failures were marked by CD4+ T cell counts persistently below 100 cells/μl (none of the patients experienced confirmed virological failure). Among all 53 patients who experienced immunological failure, 24 (45.3%) patients failed within 12 months of initial follow-up, 22 (41.5%) failed within 12 to 24 months, and 7 (13.2%) failed after 24 months. The cumulative probability of immunological failure was 18.7% at the end of 12 months, 41.4% at the end of 2 years, and 56.6% at the end of follow-up (Fig. 1A).

Characteristics of immunological failure among HIV patients are shown in Table 3. There were no differences in the ART regimens that were prescribed to patients who experienced immunological failure and patients who experienced immunological restoration. Patients who experienced immunological failure were more likely than other patients to have lower CD4+ T cell counts (z = −4.163, *P* = 0.000), lower serum sodium levels (*t* = 3.012, *P* = 0.003), higher CRP levels (*t* = −2.106, *P* = 0.038), candidiasis infections (χ^2^ = 3.053, *P* = 0.081), and be at WHO clinical stage 4 (χ^2^ = 12.856, *P* = 0.000). However, the levels of the 7 aforementioned hormones in the patients who experienced immunological failure were similar to those in the other patients. Analysis using the multivariate Cox regression and Kaplan–Meier methods that examined the 5 variables above showed that being at WHO clinical stage 4 was the only variable that associated significantly with immunological failure (HR: 3.225; 95% CI, 1.687–6.165; *P* = 0.000) (log-rank test, *P* = 0.000) (Fig. 1B and C).

**Table 3:**
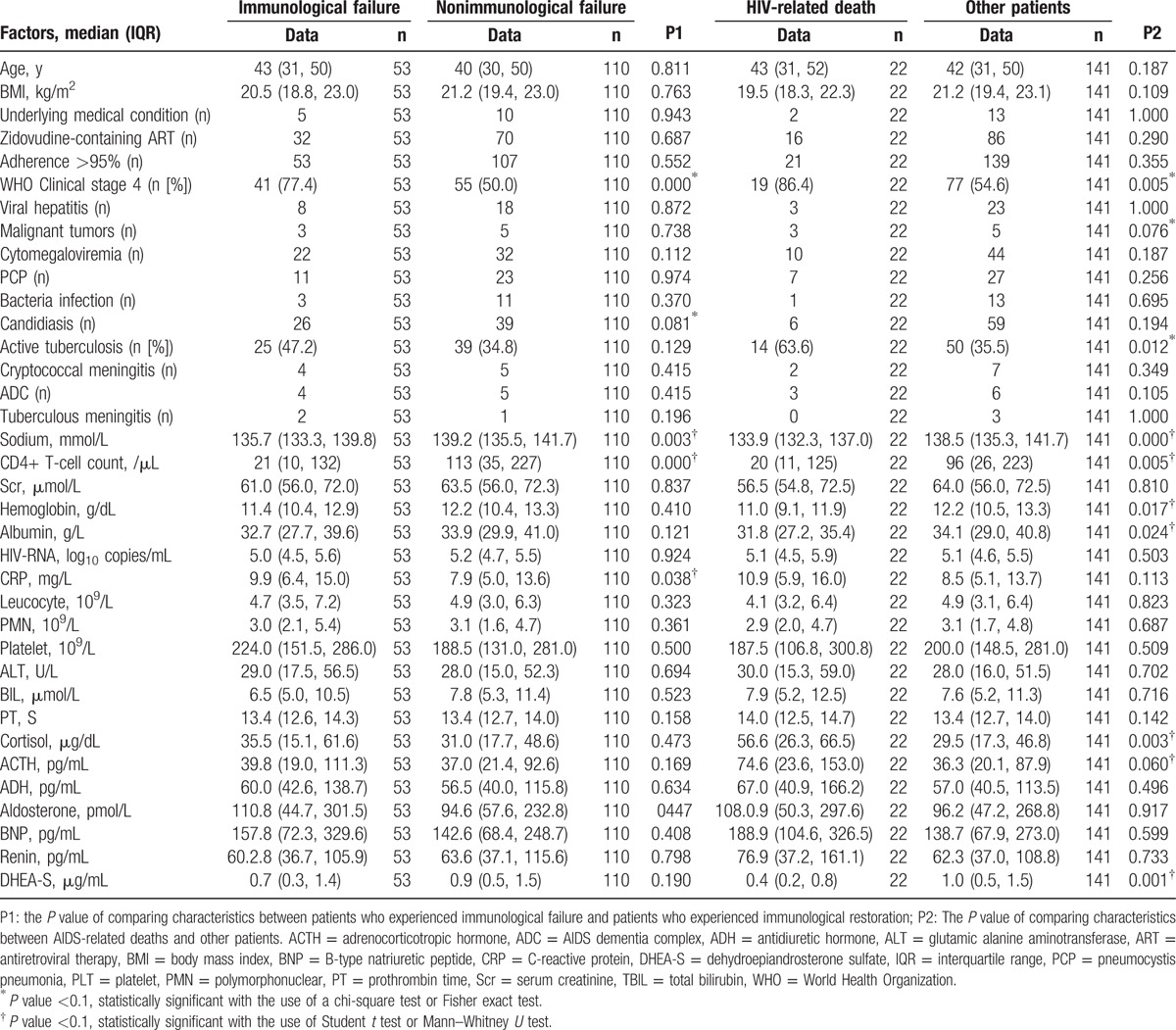
Characteristics of immunological failure and characteristics of HIV-related death among HIV patients.

### 3.4 Risk factors for mortality

**Figure 2:**
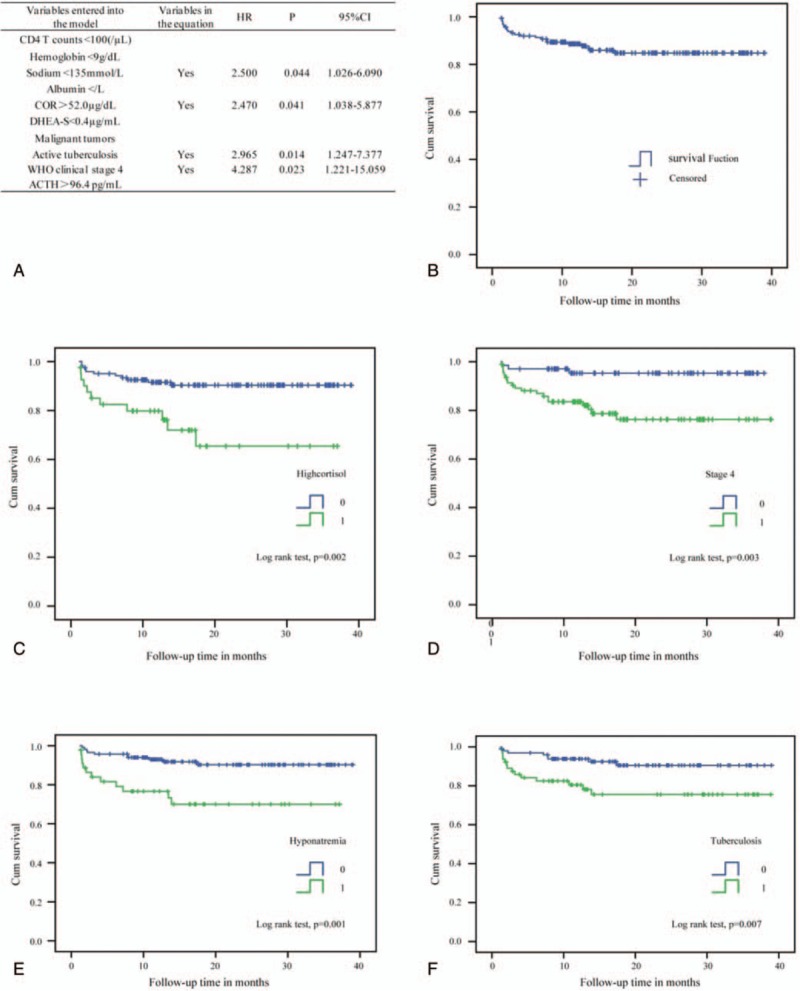
Kaplan–Meier curve and Cox regression for survival rates of human immunodeficiency virus (HIV) patients undergoing antiretroviral therapy (ART). (A) Factors associated with HIV-related death among HIV patients by multivariate Cox regression analysis. (B) Trend of cumulative survival rates of patients within 39 months of follow-up after ART. (C) High cortisol levels have a significant effect on survival rate. (D) Being at World Health Organization clinical stage 4 has a significant effect on survival rate. (E) Hyponatremia has a significant effect on survival rate. (F) Tuberculosis has a significant effect on survival rate.

Study participants were followed for a minimum of 1.13 months and a maximum of 38.97 months with total person-time follow-up of 245.33 PY. The mortality rate among the 163 HIV patients was 16.0% (26/163). Of these deaths, 84.6% (22/26) were AIDS-related, and direct causes of death were tuberculosis (10), PCP (4), cryptococcal meningitis (2), AIDS dementia complex (ADC) (2), and malignant tumors (4). Direct causes of non-AIDS-related deaths were ulcerative colitis (1 patient), cerebral hemorrhage (1 patient), aplastic anemia (1 patient), and decompensated liver cirrhosis (1 patient). Of the AIDS-related deaths, 13 patients (59.1%) died within 6 months of initial follow-up, 5 patients (22.7%) died within 6 to 12 months, and 4 patients (18.2%) died within 12 to 18 months. The cumulative probability of surviving was 91.9% after 6 months, 88.6% after 12 months, and 84.8% after 18 months (Fig. 2B).

Characteristics of HIV-related deaths among HIV patients are shown in Table 3. There were no differences between the ART regimens associated with AIDS-related deaths and the ART regimens undertaken by other patients. Regarding the variables being examined, patients whose cause of death was AIDS-related were more likely than other patients to have lower DHEA-S levels, CD4+ T cell counts, hemoglobin levels, albumin levels, and serum sodium levels (*t* = 3.349, *P* = 0.001; z = −2.805, *P* = 0.005; *t* = 2.418, *P* = 0.017; *t* = 2.279, *P* = 0.024; *t* = 4.029, *P* = 0.000), while they were also more likely to have higher cortisol and ACTH levels (*t* = −3.008, *P* = 0.003; *t* = −1.897, *P* = 0.060). However, patients whose cause of death was AIDS-related were more likely than other patients to have tuberculosis or malignant tumors and to be at WHO clinical stage 4 (χ^2^ = 6.335, *P* = 0.012; χ^2^ = 3.090, *P* = 0.076; χ^2^ = 7.926, *P* = 0.005). In analyses using the multivariate Cox regression and Kaplan–Meier methods that factored in the 10 variables mentioned above, active tuberculosis (HR: 2.965; 95% CI, 1.247–7.377; *P* = 0.014) (log-rank test, *P* = 0.007), hyponatremia (sodium <135 mmol/L) (HR: 2.500; 95% CI, 1.026–6.090; *P* = 0.044) (log-rank test, *P* = 0.001), high cortisol levels (≥52.0 μg/dL) (HR: 2.470; 95% CI, 1.038–5.877; *P* = 0.041) (log-rank test, *P* = 0.002), and being at WHO clinical stage 4 (HR: 4.287; 95% CI, 1.221–15.059; *P* = 0.023) (log-rank test, *P* = 0.003) were significantly associated with mortality (Fig. 2A, C, D, E, and F).

## 4 Discussion

Our study suggests that—apart from hyponatremia, tuberculosis, and being at WHO clinical stage 4—high cortisol levels are also an important risk factor for mortality (no collinearity was found). These findings differ from the results of a previous study that involved only 3 months of follow-up.^[16]^ Being at WHO stage 4 seems to be the only important risk factor for immunological failure. Of the patients who experienced AIDS-related deaths, 13 patients (59.1%) died within 6 months of initial follow-up, while 46 (86.8%) patients experienced immunological failure within 24 months of initial follow-up. The most important finding is that there was no significant correlation between these hormones and immunological failure, a finding that was never previously reported. Moreover, we found that DHEA-S levels have a strong correlation with both CD4+ T cell counts and WHO stages, a finding which aligns with previous findings.^[17]^

In this paper, cortisol, ACTH, BNP, and renin levels were found to be higher, while DHEA-S levels were found to be lower in HIV patients than in healthy subjects. This finding confirmed the occurrence of multiple endocrine abnormalities in HIV patients. However, our results differed from those of previous studies, which showed elevated serum DHEA-S levels during the asymptomatic stage as well as decreased cortisol and aldosterone levels.^[11,18]^ Cortisol and DHEA-S are the main circulating adrenal steroid hormones. The finding of an increase in plasma cortisol and a decrease in DHEA-S levels implies that they are important indicators for the progressive decline in immune system function during HIV infection.^[19]^ However, we did not find any correlation between cortisol levels and CD4+ T cell counts, which agrees with findings reported by Ekpebegh et al.^[20]^ High cortisol levels could be explained by severe stress,^[21,22]^ decreased cortisol catabolism,^[23]^ and alterations in the concentrations or properties of corticosteroid-binding globulin in HIV-infected patients.^[24]^ In our study, high cortisol levels could not predict immunological failure but could be a sensitive predictive marker for mortality. It is noted that hyponatremia, tuberculosis, and being at WHO stage 4 were associated with long-term mortality, a finding that is consistent with those from our previous short-term mortality study.^[25]^ Although hyponatremia is a common abnormality in patients with adrenal insufficiency, we did not find any correlation between cortisol levels and hyponatremia in this study. During severe illness, sustained hypercortisolism, as opposed to significant DHEA-S depletion, could theoretically result in an imbalance between the immunosuppressive and immunostimulatory pathways^[26]^ and, therefore, play a role in increasing susceptibility to infectious complications. We all know that basal hypocortisolemia, even if asymptomatic, should be treated with lifelong substitutive glucocorticoids, but cortisol supplementation should be reserved for only very stressful situations and, as such, is probably sufficient for patients with normal serum cortisol levels and corticotropin hyporesponsiveness. However, there is currently no recommendation for addressing hypercortisolism in HIV patients. Among HIV-infected individuals, DHEA-S is used to increase energy and muscle mass and to lessen depressive symptoms.^[27,28]^ However, recent studies have shown that ART could significantly decrease plasma cortisol levels and increase DHEA-S levels,^[29]^ which suggests that ART itself could improve hormonal disorders in HIV patients.

Although ART has substantially lowered long-term mortality rates, immunological failure is still associated with clinical failure and poor prognosis. Incomplete suppression of plasma HIV-RNA—which results from ART drug resistance, lower baseline CD4+ T cell counts, and older age—is a significant predictor of immunological failure.^[30]^ In our study, the rate of immunological failure was 32.5% and mostly occurred within 24 months of initial follow-up, which agrees with previous findings.^[31]^ We suggest that being at WHO stage 4 is a more precise indicator of severe immunodeficiency and inflammation than baseline CD4+ T cell counts are. In this study, we found that hormone concentrations had no significant impact on immunological failure. Only 18.9% patients experienced immunological failure due to virological failure resulting from ART drug resistance. Thus, further studies are needed to examine the reason for immunological failure in most patients.

It is noted that HIV itself has renin-like properties and can inappropriately alter the aldosterone:renin ratio.^[32,33]^ Circulating aldosterone concentrations were inappropriately low in patients with AIDS-related diarrhea, which is associated with profound electrolyte deficiencies and general adrenal failure.^[34]^ However, in this study, there was no difference between the aldosterone concentrations of HIV patients and those of healthy subjects. Higher levels of renin in the study's HIV patients did not correlate with HIV-RNA load, which contradicts with previous reports.^[14]^ During stressful situations, the body increases its secretion of several hormones—for example, steroids, catecholamines, growth hormone, prolactin, and vasopressin. Some papers reported that proximal tubule sodium reabsorption was preserved, while free water clearance and maximal urine dilution capability were reduced in HIV patients.^[35]^ SIADH increases total body water while decreasing total body sodium as a consequence of an enhanced or abnormal pattern of ADH release. However, we did not find any differences between the ADH or aldosterone levels of HIV patients and those of healthy subjects.

Our study has some limitations. We could not describe the improvement in hormonal disorders induced by ART itself because plasma hormone levels were only observed pre-ART without follow-up detection. In addition, the need for supplementary hormone treatment was not addressed. We did not measure corticotropin stimulation, testosterone levels, insulin secretion, or thyroid function. In addition, children, women, intravenous drug users, and former plasma donors were excluded from the study. However, in northeast China, more than 95% of patients acquire HIV infection through sexual transmission. The ratio of male-to-female HIV patients was 19:1 in our previous study. Therefore, our study is significant for physicians to clarify the characteristics of these hormones and their effect on prognosis, and our findings are compelling enough to highlight the effect of adrenal steroid hormones on mortality for patients with advanced HIV disease.

## 5 Conclusion

This study found no association between levels of common plasma hormones and immunological failure. Among endocrine abnormalities, only an abnormality in the level of baseline cortisol is a significant hormonal associated predictor of clinical mortality. The importance of intervention for these subclinical abnormalities in HIV-infected patients needs to be substantiated by a large longitudinal study.

## Acknowledgments

The authors thank the colleagues who shared their views and/or took part in discussions on the subject.
